# Synthesis of Novel Highly Functionalized 4-Thiazolidinone Derivatives from 4-Phenyl-3-thiosemicarbazones

**DOI:** 10.3390/molecules19033068

**Published:** 2014-03-11

**Authors:** Abdelmadjid Benmohammed, Omar Khoumeri, Ayada Djafri, Thierry Terme, Patrice Vanelle

**Affiliations:** 1 Laboratoire de Synthèse Organique Appliquée, BP 1524, El Menaouer, Département de Chimie, Faculté des Sciences Exactes et Appliquées, Université d’Oran, Oran 31000, Algeria; E-Mails: medmadjid@yahoo.fr (A.B.); djafriayada@yahoo.fr (A.D.); 2 Département de Sciences et Techniques, Faculté des Sciences & Technologie, Université de Mascara, Mascara 29000, Algeria; 3 Aix-Marseille Université, Institut de Chimie Radicalaire ICR, UMR CNRS 7273, Laboratoire de Pharmaco-Chimie Radicalaire, Faculté de Pharmacie, 27 Boulevard Jean Moulin, Marseille cedex 05 13385, France; E-Mails: omar.khoumeri@univ-amu.fr (O.K.); thierry.terme@univ-amu.fr (T.T.)

**Keywords:** 4-phenyl-3-thiosemicarbazones, thiazolidinones derivatives, ethyl 2-bromoacetate, diethyl acetylenedicarboxylate

## Abstract

We present herein the synthesis in good yields of two series of highly functionalized thiazolidinone derivatives from the reactions of various 4-phenyl-3-thio-semicarbazones with ethyl 2-bromoacetate and diethyl acetylenedicarboxylate, respectively.

## 1. Introduction

Thiosemicarbazones are a class of small molecules that have been evaluated over the last 50 years as antiviral [1] and as antitumoral agents [2,3,4], in addition to their antiparasitic and bacterial action against *Trypanasoma cruzi* [5,6,7] and *Toxoplasma gondii* and several bacterial strains [8]. Thiosemicarbazones have been used as intermediates for a great variety of heterocyclic products, such as thiazolidinones, thiohydantoins, thioxopyrimidinediones. It is reported that thiazolidinones exhibit antibacterial [9], antifungal [10], anticonvulsant [11], antitubercular [12], anti-inflammatory [13], antihistaminic [14,15], cardiovascular [16] and anti-HIV [17] activities. As part of our research program on new bioactive compounds [18,19,20,21,22], we report herein an efficient synthesis of some new highly functionalized thiazolidinones derived from 4-phenyl-3-thiosemicarbazones.

## 2. Results and Discussion

**Scheme 1:**
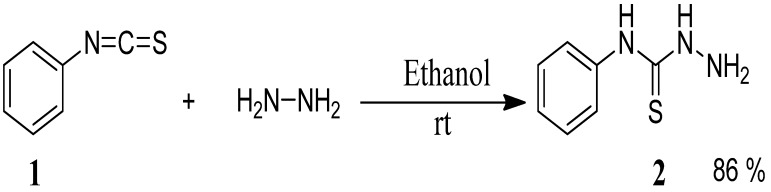
Preparation of 4-phenyl-3-thiosemicarbazide (**2**).

The starting materials, 4-phenyl-3-thiosemicarbazones **4a**–**h**, were synthesized in two steps. The first step was the preparation of 4-phenyl thiosemicarbazide (**2**) in 86% yield from phenyl isothiocyanate (**1**) and hydrazine hydrate in ethanol at room temperature [23] (Scheme 1).

**Scheme 2:**
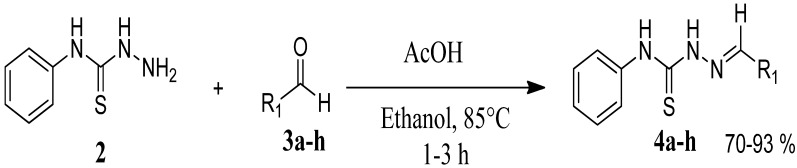
Preparation of 4-phenyl-3-thiosemicarbazones **4a**–**h**.

The reaction of 4-phenyl-3-thiosemicarbazide (**2**) with various aromatic aldehydes **3a**–**h** in the presence of few drops of acetic acid at 85 °C for 1–3 h, led to the corresponding 4-phenyl-3-thiosemicarbazone derivatives **4a**–**h** in good yields (70%–93%), as shown in Scheme 2 and Table 1.

The most characteristic signals in the ^1^H-NMR spectrum of this family of thiosemicarbazones were those corresponding to the CH=N and N-H protons. ^1^H-NMR studies showed the CH=N protons in the 7.86–8.62 ppm range, whereas thiourea N-H protons are found in the 9.13–11.78 ppm interval for N-H adjacent to the monosubstituted phenyl ring and for the N-H adjacent to the CH=N moiety, respectively. All of the synthesized compounds were in the *E*-configuration, which was confirmed using ^1^H-NMR spectroscopy, as the signal of the NH group was in the 9–12 ppm range, in comparison to the *Z*-isomer, which possesses a characteristic NH signal in the 14–15 ppm range [24].

**Table 1:** Reaction of **2** with various aromatic aldehydes **3a**–**h**.

Carbonyl compound	Product	Product number	Reaction time (h)	Yield (%)
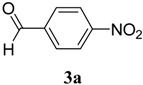	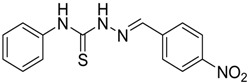	**4a**	1	70
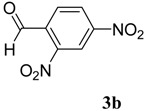	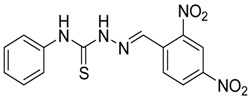	**4b**	3	90
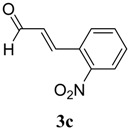	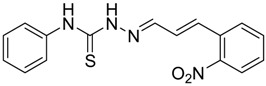	**4c**	2	93
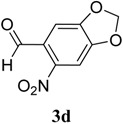	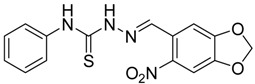	**4d**	1	90
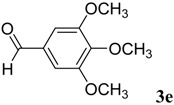	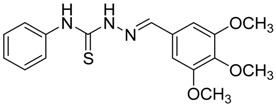	**4e**	1	91
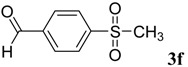	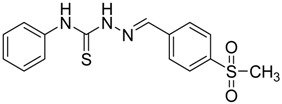	**4f**	2	89
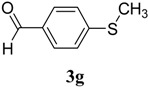	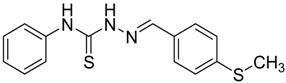	**4g**	2	91
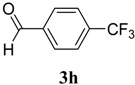	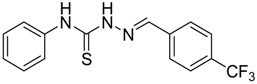	**4h**	1	89

**Scheme 3:**
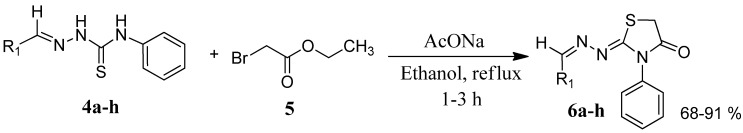
Preparation of thiazolidinones **6a**–**h**.

The reaction of various 4-phenyl thiosemicarbazones **4a**–**h** with ethyl 2-bromoacetate (**5**) as cyclizing reagent in boiling absolute ethanol containing three equivalents of anhydrous sodium acetate during 1–3 h, afforded to the thiazolidin-4-ones **6a**–**h** in good yields (68%–91%) as shown in Scheme 3 and Table 2.

**Table 2:** Reactions of **4a**–**h** with ethyl 2-bromoacetate (**5**).

Compound	Product	Product number	Reaction time (h)	Yield (%)
**4a**	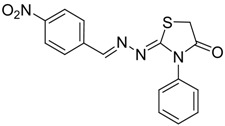	**6a**	1	91
**4b**	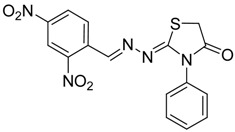	**6b**	3	68
**4c**	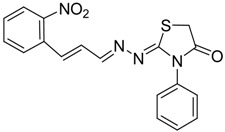	**6c**	3	82
**4d**	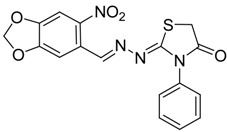	**6d**	2	86
**4e**	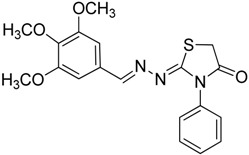	**6e**	2	80
**4f**	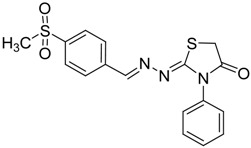	**6f**	3	89
**4g**	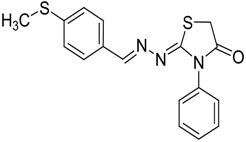	**6g**	3	90
**4h**	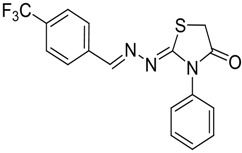	**6h**	2	88

The structures of all new compounds **6a**–**h** were established by analysis of their IR, ^1^H-NMR and ^13^C-NMR data. The IR spectra of the thiazolidin-4-ones **6a**–**h** showed absorption bands at about 1,734–1,716 cm^−1^ characteristic of (amide group) C=O stretching vibrations. Further support was obtained from the ^1^H-NMR spectra, where it did not display signs of the 4-phenyl-3-thiosemicarbazone (NH) protons. On the other hand, the ^1^H-NMR spectra exhibited resonances assigned to the SCH_2_ group of the thiazolidine ring appearing as a singlet at 3.97–4.10 ppm due to the methylene protons. The CH=N protons in these structures were observed in the 7.67–8.57 ppm region. The formation of thiazolidinones **6a**–**h** ocurred in two steps: the first step of this reaction is thought to be *S*-alkylation of thiosemicarbazide in its thiol form due to the sodium acetate used. Second step involved loss of ethanol to give the thiazolidin-4-one. The electronic and steric properties of the substituent at the 4-position of the thiosemicarbazones seems to be a determining factor for the formation of the thiazolidinone ring. Previous reports on these types of compounds reveal a small substituent such as phenyl or alkyl leads to a 4-thiazolidinone ring by loss of ethanol [25].

**Scheme 4:**
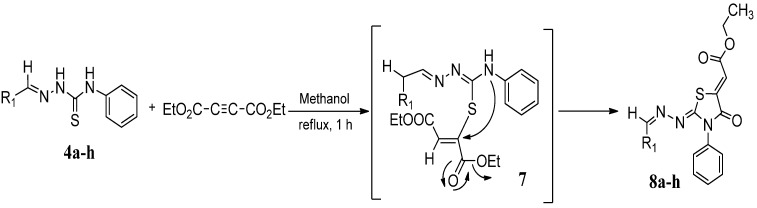
Preparation of **8a**–**h** with 4-phenyl-3-thiosemicarbazones **4a**–**h** and diethyl acetylenedicarboxylate.

The next cyclization reaction of 4-phenyl-3-thiosemicarbazones derivatives **4a**–**h** was conducted using diethyl acetylenedicarboxylate in methanol for 1 h [26], as shown in Scheme 4 and Table 3. In this reaction both of the sulfur group and the amino group are capable of reacting with diethyl acetylenedicarboxylate. It was found that the 4-phenyl thiosemicarbazone derivatives **4a**–**h** reacted with diethyl acetylenedicarboxylate exclusively with the sulfur atom. In this reaction the intermediate **7** undergoes an intramolecular cyclization which leads to the compounds **8a**–**h**.

**Table 3:** Reactions of **4a**–**h** with diethyl acetylenedicarboxylate.

Compound	Product	Product number	Yield (%)
**4a**	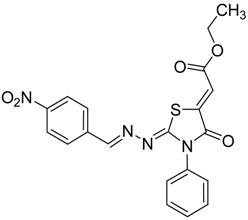	**8a**	73
**4b**	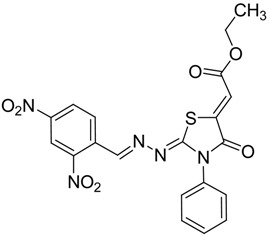	**8b**	70
**4c**	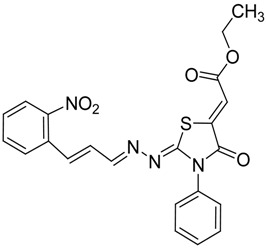	**8c**	76
**4d**	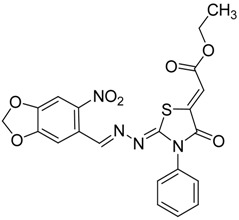	**8d**	74
**4e**	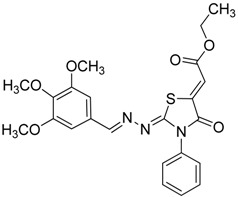	**8e**	73
**4f**	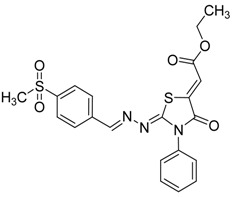	**8f**	75
**4g**	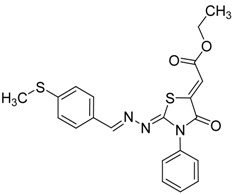	**8g**	70
**4h**	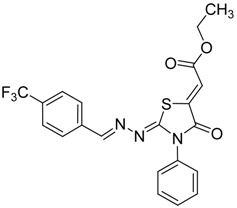	**8h**	71

**Figure 1:**
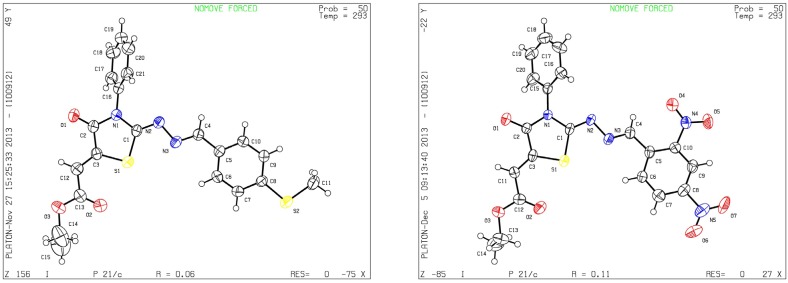
ORTEP plots of **8b** and **8g**.

Although the two geometrical *E-* or *Z-* isomers of **8a**–**h** could be formed in almost equal amounts from the reaction of diethyl acetylenedicarboxylate with **4a**–**h**, ^1^H-NMR revealed the presence of only one singlet at 6.8 ppm (vinyl proton) indicating that only one *E-* or *Z-*isomer was formed. The structures of compound **8b** and **8g** obtained by X-ray structure analysis confirmed the *Z*-configuration for the double bond in the 5-position of the thiazolidin-4-ones (Figure 1) [27,28], probably due to the steric effect of the ester group.

The chemical structures of the reaction products **8a**–**h** were confirmed by their IR, ^1^H-NMR, ^13^C-NMR spectra. The IR spectrum of compound **8a**, for example, showed absorptions at 1730, 1692 cm^−1^ due to the C=O functions of the ester and cyclic amide, respectively. Similarly, bands at 1595–1622 cm^−1^ are due to the C=N groups. The ^1^H-NMR spectrum of **8a** showed a triplet at δ = 1.28 ppm and a quartet at δ = 4.28 ppm is due to the COOCH_2_CH_3_ protons. A singlet at δ = 6.80 ppm is due to C=CH. Aromatic protons appeared as a multiplet at δ = 7.38–7.60 ppm.

## 3. Experimental

### 3.1. General

Melting points were determined on Büchi B-540 apparatus and are uncorrected. IR spectra were recorded as KBr pellets on a JASCO FT/IR4200 Fourier Transform infrared spectrometer and the reported wavenumbers are given in cm^−1^. Elemental analyses were carried out at the Spectropole, Faculté des Sciences site Saint-Jérome. ^1^H-NMR (200 MHz) and ^13^C-NMR (50 MHz) spectra were recorded on a Bruker ARX 200 spectrometer in CDCl_3_ or D_2_O at the Service Inter-Universitaire de RMN de la Faculté de Pharmacie de Marseille. The ^1^H-NMR chemical shifts were reported as parts per million downfield from tetramethylsilane (Me_4_Si), and the ^13^C-NMR chemical shifts were referenced to the solvent peaks: CDCl_3_ (76.9 ppm) or DMSO-d_6_ (39.6 ppm). Silica gel 60 (Merck, 230–400 mesh) was used for column chromatography: Thin-layer chromatography was performed with silica gel Merck 60F-254 (0.25 mm layer thickness).

### 3.2. General Procedure for the Preparation of Compounds **4a**–**h**

To a solution of 4-phenylthiosemicarbazide (**2**, 1 g, 6 mmol, 1 eq) in ethanol (33 mL) were added the benzaldehyde derivative (6.3 mmol, 1.05 eq) and acetic acid (0.50 mL). The mixture was stirred under reflux for 1–3 h and then cooled to room temperature. After, the solid separated was filtered and recrystallized from ethanol-DMF (3:1) to give compounds **4a**–**h**.

*(E)-2-(4-Nitrobenzylidene)-N-phenylhydrazinecarbothioamide* (**4a**): yellow solid; mp: 234 °C; IR (KBr) νmax/cm^−1^: 3316 and 3138 (NH), 1543 (C=N), 1292 (C=S); ^1^H-NMR (DMSO-d_6_): δ 10.32 (s, 1H, NH), 8.24 (d, 2H, *J* = 9.2 Hz, Ar-H), 8.22 (s, 1H, CH=N), 8.17 (d, 2H, *J* = 9.2 Hz, Ar-H), 7.52 (d, 2H, *J* = 7.9 Hz, Ar-H), 7.38 (dd, 2H, *J* = 7.9 Hz, *J* = 7.2 Hz, Ar-H), 7.22 ( t, 1H *J* = 7.2 Hz, Ar-H); ^13^C-NMR (DMSO-d_6_): δ 177.0 (C), 148.1 (C), 141.0 (C), 140.6 (CH), 139.4 (C), 128.9 (2CH), 128.6 (2CH), 126.7 (2CH), 126.1 (CH), 124.4 (2CH); Anal. Calcd for C_14_H_12_N_4_O_2_S: C, 55.99; H, 4.03; N, 18.65; S, 10.68. Found: C, 55.98; H, 4.06; N, 18.49; S, 10.68.

*(E)-2-(2,4-Dinitrobenzylidene)-N-phenylhydrazinecarbothioamide* (**4b**): yellow solid; mp: 219 °C; IR (KBr) ν_max_/cm^−1^: 3316 and 3138 (NH), 1543 (C=N), 1292 (C=S); ^1^H-NMR (DMSO-d_6_): δ 10.40 (s, 1H, NH), 8.87 (d, 1H, *J* = 8.8 Hz, Ar-H), 8.75 (d, 1H, *J* = 2.2 Hz, Ar-H), 8.62 (s, 1H, CH=N), 8.45 (dd, 1H, *J* = 8.8 Hz, *J* = 2.2 Hz, Ar-H), 7.52 (d, 2H, *J* = 7.7 Hz, Ar-H), 7.39 (dd, 2H, *J* = 7.7 Hz, *J* = 7.3 Hz, Ar-H), 7.24 (t, 1H, *J* = 7.3 Hz, Ar-H); ^13^C-NMR (DMSO-d_6_): δ 177.2 (C), 148.3 (C), 147.5 (C), 139.2 (C), 136.4 (CH), 134.6 (C), 130.3 (CH), 128.7 (2CH), 127.4 (CH), 126.6 (2CH), 126.3 (CH), 120.8 (CH); Anal. Calcd for C_14_H_11_N_5_O_4_S: C, 48.69; H, 3.21; N, 20.28; S, 9.29. Found: C, 48.83; H, 3.22; N, 20.08; S, 9.05.

*(E)-2-((E)-3-(2-Nitrophenyl)allylidene)-N-phenylhydrazinecarbothioamide* (**4c**): brown solid; mp: 185 °C; IR (KBr) ν_max_/cm^−1^: 3226 and 3151 (NH), 1540 (C=N), 1247 (C=S); ^1^H-NMR (CDCl_3_): δ 9.67 (s, 1H, NH), 9.14 (s, 1H, NH), 8.02 (dd, 1H, *J* = 8.2 Hz, *J* = 1.2 Hz, Ar-H), 7.76-7.59 (m, 5H, Ar-H), 7.52-7.37 (m, 4H, Ar-H), 7.25–7.29 (m, 1H, Ar-H), 6.87 (dd, 1H, *J* = 15.9 Hz, *J* = 9.2 Hz, CH); ^13^C-NMR (CDCl_3_): δ 175.6 (C), 147.8 (C), 143.3 (CH), 137.7 (C), 134.8 (CH), 133.3 (CH), 131.3 (C), 129.4 (CH), 129.0 (CH), 128.8 (2CH), 128.2 (CH), 126.2 (CH), 125.0 (CH), 124.2 (2CH); Anal. Calcd for C_16_H_14_N_4_O_2_S: C, 58.88; H, 4.32; N, 17.17; S, 9.82. Found: C, 59.13; H, 4.40; N, 17.07; S, 9.78.

*(E)-2-((6-Nitrobenzo[d][1,3]dioxol-5-yl)methylene)-N-phenylhydrazinecarbothioamide* (**4d**): yellow solid; mp: 231 °C; IR (KBr) ν_max_/cm^−1^: 3302 and 3283 (NH), 1547 (C=N), 1268 (C=S); ^1^H-NMR (DMSO-d_6_): δ 10.21 (s, 1H, NH), 8.58 (s, 1H, Ar-H), 8.17 (s, 1H, CH=N), 7.61 (s, 1H, Ar-H), 7.50 (dd, 2H, *J* = 7.8 Hz , *J* = 1.2 Hz , Ar-H), 7.37 (dd, 2H, *J* = 7.8 Hz , *J* = 7.2 Hz, Ar-H), 7.21 (td, 1H, *J* = 7.2 Hz, *J* = 1.2 Hz, Ar-H), 6.25 (s, 2H, CH_2_); ^13^C-NMR (DMSO-d_6_): δ 176.9 (C), 152.2 (C), 149.2 (C), 143.9 (C), 139.4 (C), 138.5 (CH), 128.6 (2CH), 126.94 (2CH), 126.1 (CH), 125.9 (C), 106.6 (CH), 105.3 (CH), 104.1 (CH_2_); Anal. Calcd for C_15_H_12_N_4_O_4_S: C, 52.32; H, 3.51; N, 16.27; S, 9.31. Found: C, 52.35; H, 3.46; N, 16.01; S, 9.20.

*(E)-N-Phenyl-2-(3,4,5-trimethoxybenzylidene)hydrazinecarbothioamide* (**4e**): white solide; mp: 161 °C; IR (KBr) ν_max_/cm^−1^: 3291 and 3134 (NH), 1557 (C=N), 1262 (C=S); ^1^H-NMR (CDCl_3_): δ 10.36 (s, 1H, NH), 9.14 (s, 1H, NH), 7.93 (s, 1H, CH=N), 7.63 (d, 2H, *J* = 7.8 Hz, Ar-H), 7.44 (dd, 2H, *J* = 7.8 Hz, *J* = 7.2 Hz, Ar-H), 7.30 (t, 1H, *J* = 7.2 Hz, Ar-H ), 6.90 (s, 2H, Ar-H ), 3.90 (s, 3H, OCH_3_), 3.91 (s, 6H, OCH_3_); ^13^C-NMR (CDCl_3_): δ 175.6 (C), 153.6 (2C), 143.7 (CH), 140.5 (C), 137.7 (C), 129.0 (2CH), 128.4 (C), 126.6 (CH), 125.2 (2CH), 104.7 (2CH), 61.1 (CH_3_), 56.3 (2CH_3_); Anal. Calcd for C_17_H_19_N_3_O_3_S: C, 59.11; H, 5.54; N, 12.17; S, 9.28. Found: C, 59.25; H, 5.65; N, 12.06; S, 9.19.

*(E)-2-(4-(Methylsulfonyl)benzylidene)-N-phenylhydrazinecarbothioamide* (**4f**): yellow solid; mp: 225 °C; IR (KBr) ν_max_/cm^−1^: 3325 and 3150 (NH), 1542 (C=N), 1267 (C=S); ^1^H-NMR (DMSO-d_6_): δ 10.30 (s, 1H, NH), 8.22 (s, 1H, CH=N ), 8.19 (d, 2H, *J* = 8.4 Hz, Ar-H), 7.95 (d, 2H, *J* = 8.4 Hz, Ar-H), 7.55 (d, 2H, *J* = 7.60 Hz, Ar-H), 7.39 (dd, 2H, *J* = 7.2 Hz, *J* = 7.60 Hz, Ar-H), 7.23 ( t, 1H, *J =*7.2 Hz, Ar-H), 3.26 ( s, 3H, CH_3 _); ^13^C-NMR (DMSO-d_6_): δ 177.0 (C), 141.7 (C), 141.2 (CH), 139.44 (2C), 128.7 (2CH), 128.6 (2CH), 127.7 (2CH), 126.7 (2CH), 126.10 (CH), 43.9 (CH_3_); Anal. Calcd for C_15_H_15_N_3_O_2_S_2_: C, 54.03; H, 4.53; N, 12.60; S, 19.23. Found: C, 54.11; H, 4.55; N, 12.45; S, 19.21.

*(E)-2-(4-(Methylthio)benzylidene)-N-phenylhydrazinecarbothioamide* (**4g**): yellow solid; mp: 178 °C; IR (KBr) ν_max_/cm^−1^: 3343 and 3152 (NH), 1541 (C=N), 1271 (C=S); ^1^H-NMR (CDCl_3_): δ 11.78 (s, 1H, NH), 10.08 (s, 1H, NH), 8.09 (s, 1H, CH=N), 7.82 (d, 2H, *J* = 8.4 Hz, Ar-H), 7.54 (d, 2H, *J* = 8.0 Hz, Ar-H), 7.35 (dd, 2H, *J* = 7.3 Hz, *J =* 8.0 Hz, Ar-H), 7.27 (d, *J* = 8.4 Hz, 2H, Ar-H), 7.18 (dd, 1H, *J* = 7.3 Hz, Ar-H), ^13^C-NMR (DMSO-d_6_): δ 175.4 (C), 143.1 (CH), 142.5 (C), 137.8 (C), 129.6 (C), 128.9 (2CH), 127.8 (2CH), 126.3 (CH), 125.8 (2CH), 124.8 (2CH), 15.1 (CH_3_); Anal. Calcd for C_15_H_15_N_3_O_2_S_2_: C, 59.77; H, 5.02; N, 13.94; S, 21.28. Found: C, 59.68; H, 5.04; N, 13.78; S, 21.33.

*(E)-N-Phenyl-2-(4-(trifluoromethyl)benzylidene)hydrazinecarbothioamide* (**4h**): white solid; mp: 195 °C; IR (KBr) ν_max_/cm^−1^: 3350 and 3138 (NH), 1542 (C=N), 1267 (C=S); ^1^H-NMR (CDCl_3_): δ 10.36 (s, 1H, NH), 9.18 (s, 1H, NH), 8.03 (s, 1H, CH=N), 7.85 (d, 2H, *J* = 8.1 Hz, Ar-H), 7.69–7.51 (m, 4H, Ar-H), 7.43 (d, 2H, *J* = 7.4 Hz, Ar-H), 7.29 (t, 1H, *J* = 7.3 Hz, Ar-H); ^13^C-NMR (CDCl_3_): δ 176.0 (C), 141.4 (CH), 137.5 (C), 133.9 (C), 131.5 (q, *J* = 32.9 Hz, C), 129.5 (CH), 128.9 (3CH), 127.0 (q, *J* = 3.6 Hz, CH), 126.6 (CH), 124.9 (2CH), 123.9 (q, *J* = 3.6 Hz, CH), 123.7 (q, *J* = 272.6 Hz, C); Anal. Calcd for C_15_H_12_F_3_N_3_S: C, 55.72; H, 3.74; N, 13.00; S, 9.92. Found: C, 55.79; H, 3.66; N, 12.86; S, 9.81.

### 3.3. General Procedure for the Preparation of Compounds **6a**–**h**

A mixture of compound **4a**–**h** (1.5 mmol, 1 eq), ethyl 2-bromoacetate (0.24 mL, 1.5 mmol) and anhydrous sodium acetate (0.37 g, 4.5 mmol, 3 eq) in ethanol (30 mL) was stirred until reflux; the mixture was stirred under the same conditions till the completion of the reaction (1–3 h). The reaction mixture was left to cool, poured into ice cold water, and the separated solid was ﬁltered, washed with water and recrystallized from a mixture of ethanol-DMF (3:1).

*2-((4-Nitrobenzylidene)hydrazono)-3-phenylthiazolidin-4-one* (**6a**): yellow solid; mp: 258 °C; IR (KBr) ν_max_/cm^−1^: 1716 (C=O), 1662 (C=N); ^1^H-NMR (CDCl_3_): δ 8.34 (s, 1H, CH=N), 8.25 (d, 2H, *J* = 8.8 Hz , Ar-H), 7.88 (d, 2H, *J* = 8.8 Hz , Ar-H), 7.59-7.44 (m, 3H, Ar-H), 7.35 (dd, 2H, *J* = 7.7 Hz, *J* = 1.9 Hz, Ar-H), 4.00 (s, 2H, CH_2_); ^13^C-NMR (CDCl_3_): δ 171.6 (C), 166.9 (C), 156.2 (CH), 148.9 (C), 140.0 (C), 134.3 (C), 129.4 (2CH), 129.3 (CH), 128.6 (2CH), 127.7 (2CH), 124.0 (2CH), 32.55 (CH_2_); Anal. Calcd for C_16_H_12_N_4_O_3_S: C, 56.46; H, 3.55; N, 16.46; S, 9.42. Found: C, 56.44; H, 3.5; N, 16.53; S, 9.53.

*2-((2,4-Dinitrobenzylidene)hydrazono)-3-phenylthiazolidin-4-one* (**6b**): yellow solid; mp: 275 °C; IR (KBr) νmax/cm^−1^: 1731 (C=O), 1592 (C=N); ^1^H-NMR (DMSO-d_6_): δ 8.74 (d, 1H, *J* = 2.3 Hz, Ar-H), 8.58 (dd, 1H, *J* = 8.4 Hz, *J* = 2.3 Hz , Ar-H), 8.57 (s, 1H, HC=N), 8.20 (d, 1H, *J* = 8.4 Hz, Ar-H), 7.54–7.45 (m, 3H, Ar-H), 7.41–7.36 (m, 2H, Ar-H), 4.14 (s, 2H, CH_2_); ^13^C-NMR (DMSO-d_6_): δ 172.6 (C), 170.1 (C), 152.8 (CH), 148.4 (C), 148.3 (C), 135.3 (C), 133.7 (C), 131.2 (CH), 129.4 (2CH), 129.3 (CH), 128.7 (2CH), 128.1 (CH), 120.6 (CH), 33.1 (CH_2_); Anal. Calcd for C_16_H_11_N_5_O_5_S: C, 49.87; H, 2.88; N, 18.17; S, 8.32.Found: C, 49.99; H, 2.87; N, 17.93; S, 8.25.

*2-(3-(2-Nitrophenyl)allylidene)hydrazono)-3-phenylthiazolidin-4-one* (**6c**): orange solid; mp: 265 °C; IR (KBr) ν_max_/cm^−1^: 1732 (C=O), 1627 (C=N); ^1^H-NMR (CDCl_3_): δ 8.13 (d, 1H, *J* = 9.5 Hz, CH=N), 8.0 (dd, 1H, *J* = 8.2 Hz, *J* = 1.0 Hz, Ar-H), 7.75–7.57 (m, 3H, Ar-H), 7.53–7.43 (m, 4H, Ar-H), 7.39–7.32 (m, 2H, Ar-H), 7.01 (dd, 1H, *J* = 15.8 Hz, *J* = 9.5 Hz, CH), 3.98 (s, 2H, CH_2_); ^13^C-NMR (CDCl_3_): δ 171.6 (C), 164.5 (C), 160.2 (CH), 148.00 (C), 135.8 (CH), 134.4 (C), 133.3 (2CH), 131.4 (C), 130.1 (CH), 129.4 (2CH), 129.1 (CH), 128.3 (CH), 127.7 (2CH), 125.0 (CH), 32.5 (CH_2_); Anal. Calcd for C_18_H_14_N_4_O_3_S: C, 59.01; H, 3.85; N, 15.29; S, 8.75. Found: C, 58.96; H, 3.92; N, 15.18; S, 8.50.

*2-(3,4-(Methylenedioxy)-6-nitrobenzaldehyde)hydrazono)-3-phenylthiazolidin-4-one* (**6d**): yellow solid; mp: 262 °C; IR (KBr) νmax/cm^−1^: 1717 (C=O), 1602 (C=N); ^1^H-NMR (DMSO-d_6_): δ 8.47 (s, 1H, Ar-H), 7.64 (s, 1H, HC=N), 7.56–7.44 (m, 3H, Ar-H), 7.35–7.40 (m, 1H, Ar-H), 7.36 (m, 2H, Ar-H), 6.27 (s, 2H , CH_2_), 4.10 (s, 2H, CH_2_); ^13^C-NMR (DMSO-d_6_): δ 172.5 (C), 167.7 (C), 154.0 (CH), 152.0 (C), 149.8 (C), 144.0 (C), 135.4 (C), 129.6 (2CH), 129.3 (CH), 128.7 (2CH), 125.4 (C), 106.6 (CH), 105.6 (CH), 104.4 (CH_2_), 32.9 (CH_2_); Anal. Calcd for C_17_H_12_N_4_O_5_S: C, 53.12; H, 3.15; N, 14.58; S, 8.34. Found: C, 53.15; H, 3.10; N, 14.67; S, 8.26.

*2-((3,4,5-Trimethoxybenzylidene)hydrazono)-3-phenylthiazolidin-4-one* (**6e**): white solid; mp: 174 °C; IR (KBr) νmax/cm^−1^: 1720 (C=O), 1619 (C=N); ^1^H-NMR (DMSO-d_6_): δ 8.21 (s, 1H, HC=N), 7.55–7.44 (m, 3H, Ar-H), 7.39–7.35 (m, 2H, Ar-H), 7.06 (s, 2H, Ar-H), 4.08 (s, 2H, CH_2_) , 3.78 (s, 6H, OCH_3_), 3.70 (s, 3H, OCH_3_); ^13^C-NMR (DMSO-d_6_): δ 172.6 (C), 165.4 (C), 158.0 (CH), 153.6 (2C), 140.3 (C), 135.5 (C), 130.0 (C), 129.5 (2CH), 129.1 (CH), 128.7 (2CH), 105.5 (2CH), 60.6 (CH_3_), 56.3 (2CH_3_), 32.7 (CH_2_); Anal. Calcd for C_19_H_19_N_3_O_4_S: C, 59.21; H, 4.97; N, 10.90; S, 8.32. Found: C, 59.29; H, 5.08; N, 10.64; S, 8.21.

*2-(4-(Methylsulfonyl)benzylidene)hydrazono)-3-phenylthiazolidin-4-one* (**6f**): yellow solid; mp: 277 °C; IR (KBr) ν_max_/cm^−1^: 1734 (C=O), 1615 (C=N); ^1^H-NMR (DMSO-d_6_): δ 8.43 (s, 1H, HC=N), 8.00 (d, 2H, *J* = 9.2 Hz, Ar-H), 7.94 (d, 2H, *J* = 9.2 Hz, Ar-H), 7.54–7.44 (m, 3H, Ar-H), 7.41–7.36 (m, 2H, Ar-H), 4.12 (s, 2H, CH_2_), 3.22 (s, 3H, CH_3_); ^13^C-NMR (DMSO-d_6_): δ 172.5 (C), 167.8 (C), 156.76 (CH), 142.5 (C), 139.2 (C), 135.4 (C), 129.6 (2CH), 129.2 (CH), 128.7 (4CH) , 128.0 (2CH), 43.8 (CH_3_), 32.9 (CH_2_); Anal. Calcd for C_17_H_15_N_3_O_3_S_2_: C, 54.67; H, 4.05; N, 11.25; S, 17.17. Found: C, 54.71; H, 4.05; N, 11.19; S, 17.14.

*2-(4-(Methylthio)benzylidene)hydrazono)-3-phenylthiazolidin-4-one* (**6g**): yellow solid; mp: 223 °C; IR (KBr) ν_max_/cm^−1^: 1732 (C=O), 1609 (C=N); ^1^H-NMR (CDCl_3_): δ 8.25 (s, 1H, HC=N), 7.64 (d, 2H, *J* = 8.5 Hz, Ar-H), 7.53–7.45 (m, 3H, Ar-H), 7.38–7.34 (m, 2H, Ar-H), 7.21 (d, 2H, *J* = 8.5 Hz, Ar-H), 3.97 (s, 2H, CH_2_), 2.50 (s, 3H, CH_3_); ^13^C-NMR (CDCl_3_): δ 171.8 (C), 163.9 (C), 158.4 (CH), 142.6 (C), 134.5 (C), 130.7 (C), 129.4 (2CH), 129.1 (CH), 128.4 (2CH) , 127.8 (2CH), 125.8 (2CH), 32.5 (CH_2_), 15.2 (CH_3_); Anal. Calcd for C_17_H_15_N_3_OS_2_: C, 59.80; H, 4.43; N, 12.31; S, 18.78. Found: C, 59.67; H, 4.43; N, 12.20; S, 18.88.

*2-(4-(Trifluoromethyl)benzylidene)hydrazono)-3-phenylthiazolidin-4-one* (**6h**): white solid; mp: 206 °C; IR (KBr) ν_max_/cm^−1^: 1732 (C=O), 1616 (C=N); ^1^H-NMR (CDCl_3_): δ 8.33 (s, 1H, HC=N), 7.84 (d, 2H, *J* = 8.2 Hz, Ar-H), 7.65 (d, 2H, *J* = 8.2 Hz, Ar-H), 7.59–7.43 (m, 3H, Ar-H), 7.39–7.32 (m, 2H, Ar-H), 3.99 (s, 2H, CH_2_); ^13^C-NMR (CDCl_3_): δ 171.7 (C), 165.8 (C), 157.3 (CH), 137.4 (C), 134.4 (C), 132 (q, *J* = 32.4 Hz, C), 129.4 (2CH), 129.2 (CH), 128.2 (2CH), 127.8 (3CH), 125.6 (q, *J* = 3.8 Hz, CH), 123.8 (q, *J* = 272.3 Hz, C), 32.5 (CH_2_); Anal. Calcd for C_17_H_12_F_3_N_3_OS: C, 56.19; H, 3.33; N, 11.56; S, 8.82.Found: C, 56.17; H, 3.66; N, 12.82; S, 9.96.

### 3.4. General Procedure for the Preparation of Compounds **8a**–**h**

An equimolar mixture of **4a**–**h** (1.5 mmol) and diethyl acetylenedicarboxylate (1.5 mmol) in methanol (20 mL) was refluxed for 1 h. After completion of the reaction, the reaction mixture was allowed to cool to the room temperature. The solid thus separated was collected by filtration and recrystallized using ethanol-DMF mixture.

*(Z)-Ethyl-2-(2-((4-nitrobenzylidene)hydrazono)-4-oxo-3-phenylthiazolidin-5-ylidene) acetate* (**8a**): yellow solid; mp: 206 °C; IR (KBr) ν_max_/cm^−1^: 1730 (C=O), 1692 (C=O), 1623 (C=N); ^1^H-NMR (DMSO-d_6_): δ 8.60 (s, 1H, CH=N), 8.32 (d, 2H, *J* = 8.8 Hz, Ar-H), 8.01 (d, 2H, *J* = 8.8 Hz, Ar-H), 7.58–7.48 (m, 5H, Ar-H), 6.80 (s, 1H, C=CH), 4.28 (q, 2H, *J* = 7.0 Hz, CH_2_), 1.28 (t, 3H, *J* = 7.0 Hz, CH_3_). ^13^C-NMR (DMSO-d_6_): δ 166.0 (C), 164.6 (C), 163.6 (C), 158.5 (CH), 149.2 (C), 141.7 (C), 140.0 (C), 134.5 (C), 129.6 (3CH), 129.5 (2CH), 128.6 (2CH), 124.6 (2CH), 116.2 (CH), 62.0 (CH_2_), 14.5 (CH_3_); Anal. Calcd for C_20_H_16_N_4_O_5_S: C, 56.60; H, 3.80; N, 13.20; S, 7.55. Found: C, 55.68; H, 4.59; N, 10.67; S, 5.44.

*(Z)-Ethyl-2-(2-((E)-2,4-dinitrobenzylideneamino)-4-oxo-3-phenylthiazolidin-5-ylidene) acetate* (**8b**): yellow solid; mp: 193 °C; IR (KBr) ν_max_/cm^−1^: 1727 (C=O), 1698 (C=O), 1620 (C=N); ^1^H-NMR (DMSO-d_6_): δ 8.77 (d, 1H, *J* = 2.2 Hz, Ar-H), 8.7 (s, 1H, CH=N), 8.64 (dd, 1H, *J* = 8.6 Hz, *J* = 2.2 Hz, Ar-H), 8.25 (d, 1H, *J* = 8.6 Hz, Ar-H), 7.60-7.46 (m, 5H, Ar-H), 6.83 (s, 1H, C=CH), 4.29 (q, 2H, *J* = 7.1 Hz, CH_2_), 1.28 (t, 3H, *J* = 7.1 Hz, CH_3_); ^13^C-NMR (DMSO-d_6_): δ 165.9 (C), 165.2 (C), 164.7 (C), 155.2 (CH), 148.6 (C), 141.5 (C), 134.5 (C), 133.3 (C), 131.4 (CH), 129.8 (CH), 129.7 (3CH), 128.7 (2CH), 128.3 (CH), 120.7 (CH), 116.6 (CH), 62.1 (CH_2_), 14.5 (CH_3_); Anal. Calcd for C_20_H_15_N_5_O_7_S: C, 51.17; H, 3.22; N, 14.92; S, 6.83. Found: C, 51.29; H, 3.15; N, 14.78; S, 6.77.

*(Z)-Ethyl-2-(2-((E)-3-(2-nitrophenyl)allylidene)amino)-4-oxo-3-phenylthiazolidin-5-ylidene) acetate* (**8c**): yellow solid; mp: 251 °C; IR (KBr) ν_max_/cm^−1^: 1727 (C=O), 1692 (C=O), 1618 (C=N); ^1^H-NMR (DMSO-d_6_): δ 8.27 (d, 1H, *J* = 9.4 Hz, CH), 8.01 (ddd, 2H, *J* = 7.1 Hz, *J* = 7.9 Hz, *J* = 1.1 Hz, Ar-H), 7.73 (dd, 1H, *J* = 7.9 Hz, *J* = 7.1 Hz, Ar-H), 7.63-7.42 (m, 7H, Ar-H), 7.20 (dd, 1H, *J* = 15.7 Hz, *J* = 9.4 Hz, Ar-H), 6.77 (s, 1H, C=CH), 4.27 (q, 2H, *J* = 7.1 Hz, CH_2_), 1.27 (t, 3H, *J* = 7.1 Hz, CH_3_); ^13^C-NMR (DMSO-d_6_): δ 166.0 (C), 164.6 (C), 162.1 (CH), 161.5 (C), 148.6 (C), 141.9 (C), 137.3 (CH), 134.6 (C), 134.0 (CH), 130.7 (CH), 130.4 (C), 129.8 (CH), 129.6 (3CH), 129.0 (CH), 128.6 (2CH), 125.0 (CH), 115.9 (CH), 62.0 (CH_2_), 14.5 (CH_3_). Anal. Calcd for C_22_H_18_N_4_O_5_S: C, 58.66; H, 4.03; N, 12.44; S, 7.12. Found: C, 58.47; H, 3.92; N, 12.05; S, 6.64

*(Z)-Ethyl-2-(2-((E)-(4-nitrobenzo[d][1,3]dioxol-5-yl)methyleneamino)-4-oxo-3-phenylthiazolidin-5-ylidene) acetate* (**8d**): yellow solid; mp: 251 °C; IR (KBr) ν_max_/cm^−1^: 1721 (C=O) 1702 (C=O), 1597 (C=N); ^1^H-NMR (DMSO-d_6_): δ 8.59 (s, 1H, Ar-H), 7.70 (s, 1H, Ar-H), 7.54–7.47 (m, 5H, Ar-H), 7.38 (s, 1H, Ar-H), 6.80 (s, 1H, C=CH), 6.30 (s, 2H, CH_2_ ), 4.27 (q, 2H, *J* = 7.1 Hz, CH_2_), 1.27 (t, 3H, *J* = 7.1 Hz, CH_3_); ^13^C-NMR (DMSO-d_6_): δ 166.0 (C), 164.7 (C), 163.0 (C), 156.5 (CH), 152.1 (C), 150.3 (C), 144.3 (C), 141.8 (C), 137.2 (C), 134.6 (C), 129.7 (2CH), 128.7 (2CH), 124.9 (CH), 116.2 (CH), 106.8 (CH), 105.8 (CH), 104.6 (CH_2_), 62.0 (CH_2_), 14.5 (CH_3_); Anal. Calcd for C_21_H_16_N_4_O_7_S: C, 53.84; H, 3.44; N, 11.96; S, 6.85. Found: C, 53.79; H, 3.42; N, 11.98; S, 6.75.

*(Z)-Ethyl-2-(2-((E)-3,4,5-trimethoxybenzylideneamino)-4-oxo-3-phenylthiazolidin-5-ylidene) acetate* (**8e**): yellow solid; mp: 145 °C; IR (KBr) ν_max_/cm^−1^: 1721 (C=O), 1689 (C=O), 1622 (C=N); ^1^H-NMR (DMSO-d_6_): δ 8.35 (s, 1H, CH=N), 7.58–7.47 (m, 5H, Ar-H), 7.11 (s, 2H, Ar-H), 6.77 (s, 1H, C=CH), 4.27 (q, 2H, *J* = 7.0 Hz, CH_2_), 3.80 (s, 6H, OCH_3_), 3.70 (s, 3H, OCH_3_), 1.28 (t, 3H, *J* = 7.0 Hz, CH_3_); ^13^C-NMR (DMSO-d_6_): δ 165.9 (C), 164.6 (C), 160.6 (C), 160.4 (CH), 153.6 (2C), 142.0 (C), 140.9 (C), 134.7 (C), 129.6 (3CH), 129.4 (C), 128.6 (2CH), 115.7 (CH), 105.9 (2CH), 62.0 (CH_2_), 60.6 (CH_3_), 56.3 (2CH_3_), 14.5 (CH_3_); Anal. Calcd for C_23_H_23_N_3_O_6_S: C, 58.84; H, 4.94; N, 8.95; S, 6.83. Found: C, 58.47; H, 4.93; N, 8.77; S, 6.64.

*(Z)-Ethyl-2-(2-((E)-4-(methylsulfonyl)benzylideneamino)-4-oxo-3-phenylthiazolidin-5-ylidene) acetate* (**8f**): yellow solid; mp: 254 °C; IR (KBr) νmax /cm^−1^: 1722 (C=O), 1687 (C=O), 1611 (C=N); ^1^H-NMR (DMSO-d_6_): δ 8.58 (s, 1H, CH=N), 8.02 (s, 4H, Ar-H), 7.59–7.45 (m, 5H, Ar-H), 6.81 (s, 1H, C=CH), 4.29 (q, 2H, *J* = 7.1 Hz, CH_2_), 3.24 (s, 3H, CH_3_), 1.28 (t, 3H, *J* = 7.1 Hz, CH_3_); ^13^C-NMR (DMSO-d_6_): δ 166.0 (C), 164.7 (C), 163.2 (C), 159.0 (CH), 143.0 (C), 141.8 (C), 138.6 (C), 134.6 (C), 129.7 (3CH), 129.1 (2CH), 128.7 (2CH), 128.1 (2CH), 116.1 (CH), 62.1 (CH_2_), 43.9 (CH_3_), 14.5 (CH_3_); Anal. Calcd for C_21_H_19_N_3_O_5_S_2_: C, 55.13; H, 4.19; N 9.18; S, 14.02. Found: C, 55.18; H, 4.19; N, 8.96; S, 14.11.

*(Z)-Ethyl-2-(2-((E)-4-(methylthio)benzylideneamino)-4-oxo-3-phenylthiazolidin-5-ylidene)acetate* (**8g**): yellow solid; mp: 210 °C; IR (KBr) ν_max_/cm^−1^: 1720 (C=O), 1696 (C=O), 1604 (C=N); ^1^H-NMR (DMSO-d_6_): δ 8.40 (s, 1H, CH=N), 8.70 (d, 2H, *J* = 8.4 Hz, Ar-H), 7.58–7.44 (m, 5H, Ar-H), 7.33 (d, 2H, *J* = 8.4 Hz, Ar-H), 6.77 (s, 1H, C=CH), 4.28 (q, 2H, *J* = 7.1 Hz, CH_2_), 3.31 (s, 3H, CH_3_), 1.28 (t, 3H, *J* = 7.1 Hz, CH_3_); ^13^C-NMR (DMSO-d_6_): δ 166.0 (C), 164.6 (C), 160.9 (C), 160.0 (CH), 143.5 (C), 142.1 (C), 134.7 (C), 130.3 (C), 129.6 (3CH), 128.9 (2CH), 128.7 (2CH), 126.0 (2CH), 115.6 (CH), 62.0 (CH_2_), 14.6 (CH_3_), 14.5 (CH_3_); Anal. Calcd for C_21_H_19_N_3_O_3_S_2_: C, 59.27; H, 4.50; N 9.87; S, 15.07. Found: C, 59.10; H, 4.48; N, 9.74; S, 14.86.

*(Z)-Ethyl-2-(2-((E)-4-(trifluoromethyl)benzylideneamino)-4-oxo-3-phenylthiazolidin-5-ylidene) acetate* (**8h**): yellow solid; mp: 154 °C; IR (KBr) ν_max_/cm^−1^: 1721 (C=O), 1690 (C=O), 1619 (C=N); ^1^H-NMR (DMSO-d_6_): δ 8.56 (s, 1H, CH=N), 8.00 (d, 2H, *J* = 8.2 Hz, Ar-H), 7.84 (d, 2H, *J* = 8.2 Hz, Ar-H), 7.58-7.45 (m, 5H, Ar-H), 6.80 (s, 1H, C=CH), 4.30 (q, 2H, *J* = 7.1 Hz, CH_2_), 1.28 (t, 3H, *J* = 7.1 Hz, CH_3_); ^13^C-NMR (DMSO-d_6_): δ 166.0 (C), 164.6 (C), 162.9 (C), 159.1 (CH), 141.9 (C), 137.9 (C), 134.6 (C), 131.3 (q, *J* = 32.2 Hz, C), 129.6 (q, *J* = 272.2 Hz, C), 129.5 (3CH), 129.1 (2CH), 128.7 (2CH), 126.3 (q, *J* = 3.2Hz, 2CH), 116.0 (CH), 62.0 (CH_2_), 14.5 (CH_3_); Anal. Calcd for C_21_H_16_F_3_N_3_O_3_S: C, 56.37; H, 3.60; N, 9.39; S, 7.17. Found: C, 56.33; H, 3.51; N, 9.17; S, 7.10.

## 4. Conclusions

In conclusion, we have prepared a series of 4-phenyl-3-thiosemicarbazone derivatives from 4-phenyl-3-thiosemicarbazide and various aromatic aldehydes substituted with different electron-donor and -withdrawing groups. In a second step, these 4-phenyl-3-thiosemicarbazone derivatives were reacted with 2-ethyl bromoacetate and diethyl acetylenedicarboxylate, respectively, to afford an original series of highly functionalized thiazolidinone derivatives in good yields. The antiparasitic and antibacterial evaluations of all synthesized compound are under investigation.
